# Facial morphology and obstructive sleep apnea

**DOI:** 10.1590/2177-6709.20.6.060-067.oar

**Published:** 2015

**Authors:** Anderson Capistrano, Aldir Cordeiro, Leopoldino Capelozza, Veridiana Correia Almeida, Priscila Izabela de Castro e Silva, Sandra Martinez, Renata Rodrigues de Almeida-Pedrin

**Affiliations:** 1 Professor of Occlusion and Orthodontics, Faculdade de Odontologia de Recife (FOR-PE), Recife, Pernambuco, Brazil; 2 MSc in Orthodontics, Universidade Sagrado Coração (USC), Bauru, São Paulo, Brazil; 3 Professor, Universidade Sagrado Coração (USC), Undergraduate and Graduate programs, Bauru, São Paulo, Brazil; 4 Postgraduate student, Cooperativa dos Odontologistas de Pernambuco (COOPE), Recife, Pernambuco, Brazil; 5 Student, Sindicato dos Odontologistas no Estado de Pernambuco (SOEPE), Postgraduate program in Orthodontics, Recife, Pernambuco, Brazil; 6 Specialist in Sleep Medicine, Universidade de São Paulo (USP), São Paulo, São Paulo, Brazil, and Stanford University, Stanford, California, USA

**Keywords:** Diagnosis, Face, Obstructive sleep apnea

## Abstract

**Objective:**

This study aimed at assessing the relationship between facial morphological patterns (I, II, III, Long Face and Short Face) as well as facial types (brachyfacial, mesofacial and dolichofacial) and obstructive sleep apnea (OSA) in patients attending a center specialized in sleep disorders.

**Methods:**

Frontal, lateral and smile photographs of 252 patients (157 men and 95 women), randomly selected from a polysomnography clinic, with mean age of 40.62 years, were evaluated. In order to obtain diagnosis of facial morphology, the sample was sent to three professors of Orthodontics trained to classify patients' face according to five patterns, as follows: 1) Pattern I; 2) Pattern II; 3) Pattern III; 4) Long facial pattern; 5) Short facial pattern. Intraexaminer agreement was assessed by means of Kappa index. The professors ranked patients' facial type based on a facial index that considers the proportion between facial width and height.

**Results:**

The multiple linear regression model evinced that, when compared to Pattern I, Pattern II had the apnea and hypopnea index (AHI) worsened in 6.98 episodes. However, when Pattern II was compared to Pattern III patients, the index for the latter was 11.45 episodes lower. As for the facial type, brachyfacial patients had a mean AHI of 22.34, while dolichofacial patients had a significantly statistical lower index of 10.52.

**Conclusion:**

Patients' facial morphology influences OSA. Pattern II and brachyfacial patients had greater AHI, while Pattern III patients showed a lower index.

## Abstract (pt)

**Objetivo:**

o objetivo deste estudo foi avaliar a associação entre os padrões morfológicos faciais e tipos faciais (braquifacial, mesofacial e dolicofacial) com a Apneia Obstrutiva do Sono (AOS) em pacientes de um centro especializado em distúrbios do sono.

**Métodos:**

foram utilizadas fotografias faciais de frente, perfil e sorriso de 252 indivíduos, selecionados aleatoriamente entre pacientes que procuraram uma clínica especializada em polissonografia. Para o diagnóstico morfológico facial, a amostra foi enviada a três professores de Ortodontia treinados na classificação do padrão facial, e cada um recebeu a orientação para classificar o padrão facial da seguinte forma: 1 = Padrão I, 2 = Padrão II, 3 = Padrão III, 4 = Padrão Face Longa e 5 = Padrão Face Curta. A concordância interexaminadores foi avaliada por meio do Índice Kappa. O diagnóstico do tipo facial foi estabelecido por meio de um índice facial que leva em consideração a proporção entre a largura e altura da face.

**Resultados:**

no modelo de regressão linear múltipla, ficou evidenciado que o Padrão II teve a capacidade de agravar o índice de apneia e hipopneia (IAH) em 6,98, enquanto os pacientes do Padrão III tinham esse índice atenuado em 11,45. Para o tipo facial, os pacientes braquifaciais apresentaram um IAH médio de 22,34, enquanto o grupo classificado como dolicofacial mostrou um índice menor, de 10,52, com significância estatística.

**Conclusão:**

o desenho morfológico facial se mostrou um considerável fator de agravamento ou proteção da SAOS, onde os indivíduos Padrão II e braquifaciais tiveram IAH maiores, enquanto nos pacientes Padrão III esse índice foi reduzido.

## INTRODUCTION

In the last 20 years, Dentistry has discussed cases of snoring and obstructive sleep apnea (OSA). Within an interdisciplinary approach, neurologists, otolaryngologists, physiotherapists, speech therapists and clinicians have all recognized the importance of assessing these patients from the point of view of Dentistry, not only in terms of therapeutic control, but also in preventing it by means of treating potential malocclusions that could increase the risk of airway disorders, particularly when they are associated with predisposing factors such as obesity, hypertension and aging.^1^

Obstructive sleep apnea (OSA) is a common disorder associated with snoring, upper airway collapse at sleep, oxygen desaturation and fragmented sleep. It is also associated with cardiovascular morbidity, risk of car accidents and general mortality.^1^ OSA diagnosis is not simple, since polysomnography requires patient's monitoring by a specialist at a sleep laboratory, which renders the procedure relatively expensive and difficult. In order to simplify diagnosis, a study aimed at assessing differences in craniofacial phenotype among caucasian patients with OSA. The study found that detailed anatomic data, such as facial width, distance between eyes, as well as mandibular length and chin-neck angle, were useful in predicting OSA with a sensitivity index of 86%.^2^

Anatomical airway narrowing is among the etiological factors of snoring and OSA. It consists in soft tissues excess, macroglossia and retrognathism. This condition causes great resistance that hinders air flow and engenders negative intraluminal pressure while inspiring, thereby favoring breathing collapse. The risk of developing this disorder significantly increases with weight gain, aging, increased neck circumference and alcohol consumption. The following systemic conditions also appear as predisposing factors: systemic hypertension, untreated hypothyroidism, acromegaly and nasal obstruction.^3^

OSA recognition in the overall population remains low and most patients are not diagnosed.^1^ Thus, there is a critical, clinical need to develop better methods that allow OSA recognition and diagnosis. The present study was conducted to assess the relationship between facial morphological pattern, within a contemporary context of genetic determinism, and OSA. This relationship might stand for an important clinical evidence of quick, inexpensive and simple diagnosis of a disease that significantly impairs patients' quality of life.

The literature^2^^-^ 7 has demonstrated a relationship between craniofacial dimensions and upper airway structures in patients with OSA. These results give support to the potential role facial measurements play in the anatomical phenotype of OSA.

 Craniofacial morphology seems to be among the predisposing factors for the development of OSA. Mandibular deficiency and increased anterior-inferior facial height highlight such possibility.^7^ The majority of publications^7^^-^ 12 used cephalometric measurements to define craniofacial morphology, which may cause considerable doubt and contradiction. In an attempt to avoid it, the present study aims at assessing a diagnosis system that considers the genetic determinism of craniofacial morphology, with specific methods centered around a diagnosis concept based on facial patterns,^13^^,^ 14 in addition to studying its relationship with obstructive sleep apnea in adult patients.

## MATERIAL AND METHODS

### Sample calculation

For sample size calculation, alpha = 5% and the power of the test was of 80%. Calculation was based on multiple linear regression analysis, with AHI as the dependent variable; and sex, age, BMI, facial type (three categories) and facial pattern (five categories) as independent variables, thereby totaling 11 predictor variables. Effect size was set at 0.10 and minimal sample size was of 178 cases. Calculation was carried out using the software developed by Soper,^15^ in 2014.

### Sample selection

The final sample comprised 252 patients with a mean age of 40.62 (from 18 to 62 years old) and a mean BMI of 28.74 ± 4.73). A total of 157 men and 95 women who were referred to a center specialized in sleep disorders and polysomnography. Patients' main complaint involved snoring, insomnia, restless nights, chronic pain, memory deficit, bruxism and daytime sleepiness. The research project from which the present study originated was approved by Universidade Sagrado Coração (USC) Institutional Review Board under protocol #412.260. All patients signed an informed consent form.

After interviewing an average of 900 patients, some of them were excluded based on the following criteria:

» BMI over 40.
» Impaired posterior occlusal support.
» Patients with a beard that hindered facial analysis.
» Patients using continuous positive airway pressure (CPAP) or intraoral appliance and who were being subjected to the examination, so as to assess the therapeutic effectiveness of the devices.
» Craniofacial syndromic patients.
» Patients with chronic obstructive pulmonary diseases (CPOC) or neurological or mental disorders who were affected by upper airway infection.
» Patients with history of orthognathic surgery or any other type of airway surgery.
» Patients under 18 and above 62 years old.
» Patients who, for any reason, did not agree in taking part in the research.
» After facial pattern analysis, other patients were also excluded for presenting three different diagnoses.

The 252 individuals comprising the sample were divided into two groups according to polysomnography results. The group without OSA (Group I, 77 patients) and the group with OSA (Group II, 175 patients) which presented an AHI value greater than five episodes of apnea per hour of sleep, enough to characterize the individuals as having the disease.

**Figure 1:**
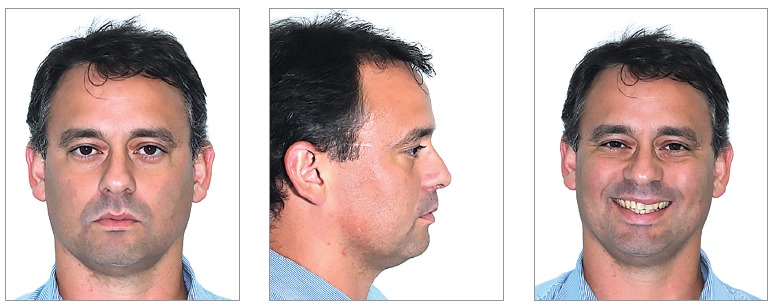
- Example of photograph set-up for diagnosis.

Frontal, lateral and smile standardized photographs were used to assess patients' facial morphological pattern (Fig 1). The photographs were inserted in a PowerPoint^TM^ slide presentation and sent via WeTransfer^TM^ to three experienced orthodontic professors. Each examiner was advised to classify patients' facial pattern according to the following: 1) Pattern I; 2) Pattern II; 3) Pattern III; 4) Long Facial Pattern; 5) Short Facial Pattern. Examiners did not have access to patients' reports and, for this reason, were unaware of those with and without OSA.

**Figure 2:**
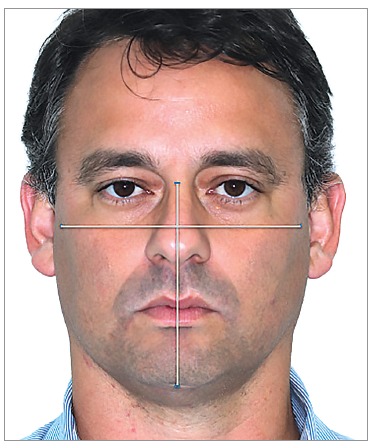
- One of the patients comprising the sample. Image used to illustrate how facial type was obtained.

Examiners reached facial type diagnosis through a facial index (n-gn/zy-zy)^16^ that considers the proportion between facial width and height, with a mean value of 88.5 for men and 86.2 for women (Table 1). Measurements were obtained with the aid of Photoshop^TM^ CS4 software (Fig 2).

**Table 1:** - Facial index.

	**Men**	**Women**
Mesofacial	83.4 - 93.6	81.6 - 90.8
Brachyfacial	< 83.4	< 81.6
Dolichofacial	> 93.6	> 90.8

Patients were classified as mesofacial, brachyfacial and dolichofacial, as shown in Table 1. 

### Error of the method

To assess the error of classifying both facial pattern and type, Kappa index^17^ was used (Table 2). For facial pattern, the diagnoses of three examiners were crossed for each patient. Patients with three different diagnoses were excluded from this study. Four patients were excluded by means of this criterion. As for facial type, 30% of the sample was randomly selected, so as to reassess patients' facial proportions after 30 days.

**Table 2:** - Agreement percentage and Kappa's values for intraexaminer agreement assessment.

**Measurement**	**% of agreement**	**Kappa**
Examiner 1 *versus* Examiner 2	77.8	0.64
Examiner 1 *versus* Examiner 3	77.4	0.62
Examiner 2 *versus* Examiner 3	81.4	0.68
Facial type 1 *versus* Facial type 2	90.4	0.90

All assessments reached an agreement value that ranged between strong and nearly perfect. Landis & Koch's classification^18^ was used as reference.

### Statistical analysis

Data were arranged in tables and charts using absolute (n) and relative (%) frequencies. Quantitative variables were described by mean and standard deviation parameters.

To assess the relationship between variables, Kruskal-Wallis, chi-square and Spearman's correlation tests were used.

To assess the combined effect of sex, age, BMI, facial type and facial pattern on the AHI value, stepwise backward multiple linear regression analysis was used. Significance level was set at 5% (*p* < 0.05) for all tests. All statistical procedures were carried out by means of Statistica version 12 (StatSoft Inc., Tulsa, USA) software.

## RESULTS

Of the 289 patients, 29 were excluded based on the exclusion criteria. A total of 260 patients were analyzed, four of which were excluded for presenting three different morphological diagnoses, one for not having polysomnography concluded and three for not having polysomnography results sent for analysis within a reasonable time. Thus, 252 patients were included in the statistical analysis.

In terms of facial morphological pattern and OSA prevalence, patients were classified according to data presented in Table 3. Data analysis revealed statistical difference between Pattern II and long face. Despite no relevant statistically significant difference, the percentage of short face individuals with OSA is greater than expected, as it totaled 77.8% against 22.2% for individuals without the disorder. Both percentages are significantly near those found for Pattern II, 80.3% and 19.7%, respectively. When facial patterns were assessed by Kruskal-Wallis test, according to the absolute AHI value, there was statistically significant difference between Pattern II and long face (Table 4). Importantly, it is worth noting that the mean AHI value for the Pattern III group (11.4) was nearly half that of the Pattern II group (22.51).

**Table 3:** - Relationship between facial pattern and OSA.

**Facial pattern**	**Group I**	**Group II**	**Total**		
	**n**	**%**	**n**	**%**	
Pattern I	46	32.6	95	67.4	141
Pattern II	15	19.7	61	80.3	76
Pattern III	7	43.8	9	56.3	16
Short face	2	22.2	7	77.8	9
Long face	7	70.0	3	30.0	10

Chi-square (*p* = 0.009* - Pattern II ≠ Long Face).

**Table 4:** - Comparison among the five facial patterns with regard to AHI measurements based on Kruskal-Wallis test.

**Facial pattern**	**n**	**Mean**	**SD**	***p*-value**
Pattern I	141	17.97	21.4	
Pattern II	76	22.51	23.05	0.027 (P II ≠ L.F.)
Pattern III	16	11.4	13.31	
Short face	9	16.03	16.31	
Long face	10	6.22	9.94	

Table 5 shows no statistically significant differences for distribution of facial types for groups I and II. Nevertheless, when groups were classified according to the AHI value (Table 6), there was statistically significant difference between brachyfacial patients with a mean AHI of 22.34, and dolichofacial patients with a mean AHI value of 10.52.

**Table 5:** - Relationship between facial type and OSA.

**Facial type**	**Without OSA**	**With OSA**	**Total**		
	**n**	**%**	**n**	**%**	
Mesofacial	44	32.6	91	67.4	135
Brachyfacial	26	26.5	72	73.5	98
Dolichofacial	7	36.8	12	63.2	19

Chi-square *(p* = 0.505 ns).

**Table 6:** - Comparison among the three facial types with regard to AHI measurements based on Kruskal-Wallis test.

**Facial type**	**n**	**Mean**	**SD**	***p*-value**	
Mesofacial	135	16.63	19.51		
Brachyfacial	98	22.34	23.87	0.044*	B ≠ D
Dolichofacial	19	10.52	14.65		

Results reveal that facial pattern influenced the apnea and hypopnea index (AHI) severity when multiple linear regression analysis was used. This is because apnea is a multifactorial disorder in which the interaction among variables, such as BMI, sex and age, also influence AHI values. Importantly, multiple linear regression analysis is considered adequate for data analysis, since it allows assessment of interaction between two or among more than two variables.

Table 7 shows that when using Pattern I as a pattern of comparison, Pattern II had AHI severity worsened in 6.98 episodes. Conversely, Table 8 shows that, when Pattern II was used as reference, Pattern I individuals had the AHI value reduced in 5.06 while Pattern III patients had the AHI reduced in 11.45.

**Table 7:** - Stepwise backward multiple linear regression analysis with AHI as dependent variable; and sex, age, BMI, facial type and facial pattern as independent variables.

**Independent variables**	**B**	**B pattern error**	**Beta**	***p*-value**	**Adjusted R^2^**	***p*-value**
Constant	-49.50	7.97		<0.001*	0.28	<0.001*
Sex 0 = F; 1 = M	10.953	2.406	0.250	<0.001*		
Age	0.524	0.100	0.287	<0.001*		
BMI	1.311	0.246	0.292	<0.001*		
Pattern II P. I = 0	6.983	2.548	0.151	0.007*		

**Table 8:** - Stepwise backward multiple linear regression analysis with AHI as dependent variable; and sex, age, BMI, facial type and facial pattern as independent variables.

**Independent variables**	**B**	**B pattern error**	**Beta**	***p*-value**	**Adjusted R^2^**	***p*-value**
Constant	-44.76	7.84		<0.001*	0.26	<0.001*
Sex: 0 = F; 1 = M	11.349	2.402	0.259	<0.001*		
Age	0.530	0.101	0.291	<0.001*		
BMI	1.326	0.248	0.295	<0.001*		
Pattern I P. II = 0	-5.056	2.495	-0.118	0.044*		
Pattern III P. II = 0	-11.453	4.950	-0.132	0.021*		

* - statistically significant (*p* < 0.05).

As shown in Tables 7 and 8, beta analysis reveals that the following factors influenced AHI in ascending order: facial morphological pattern, male sex, age and BMI.

## DISCUSSION

Craniofacial morphology plays an important role in OSA^2^^,^ 4 ^-^ 7 in adult patients. Increased gonial angle, changes in anterior and posterior facial height, decreased anterior cranial base and mandibular deficiency seem to contribute to pharyngeal airway narrowing.^7^

Cephalometry is, without a doubt, the method most used in current literature to analyze facial morphology and its relationship with OSA.^7^^-^ 12 Nevertheless, with a view to rendering diagnosis easier, or at least suspecting the existence of the disease, front-view and profile photographs have been used to assess anatomical data, such as facial width, distance between the eyes, chin-neck angle and mandibular length.^2^^,^ 4

Morphological expression used for diagnosis in Orthodontics might be better assessed and understood by means of facial soft tissues analysis. In this sense, cephalometry plays a secondary and supplementary role and could not be used as a primary diagnostic tool to determine facial morphology. The reproducibility and reliability of the method still allow assessment of the influence of growth and therapeutic actions on facial morphology.^3^^,^ 4

Analysis of facial morphology was based on the clinical experience of three professors of Orthodontics. Intraexaminer agreement yielded good results (78.9%) with a Kappa index of 0.65 (strong agreement). The methods employed in the present study were based on Reis et al^19^ who found an intraexaminer agreement of 72%, with Kappa index of 0.65 (strong agreement). Both values were close to those found for the present study. With a view to rendering the relationship between facial morphology, assessed within a subjective approach, and OSA, four individuals were excluded from the sample due to having three different diagnoses.

A total of 30% of the sample was randomly selected and measured again after 30 days, so as to reassess patients' facial proportions. Agreement was of 90.4% with a Kappa index of 0.90 (nearly perfect), thereby resulting in high reliability of research results.

Due to lack of studies focusing on establishing a relationship between facial morphology and obstructive sleep apnea, based on the subjective determination of facial morphological pattern and facial type as well as analysis of proportion between facial width and height, comparison of results is usually made with facial anthropometric measurements and, most of times, by means of cephalometry.

One of the most common findings in the literature addressing patients' facial morphology is the relationship established between OSA and convex facial profile,^12^^,^ 15 ^,^ 20 ^,^ 21 even though Katyal et al's systematic review^8^ minimizes such association, at least in children. Although no statistically significant difference was found, Pattern II patients present greater OSA prevalence (80.3%) in comparison to patients without the disorder (19.7%). In terms of AHI severity, Pattern II individuals present the greatest incidence, with 22.51 episodes of apnea per hour of sleep in comparison to 11.40 episodes for Pattern III individuals who are morphologically opposite to Pattern II.^13^ These results reveal a tendency of OSA worsening in Pattern II patients, even though statistically significant difference was only observed when the Pattern II group was compared to Long Face patients.

As for patients' facial type, Table 6 shows that brachyfacial individuals with a mean AHI value of 22.34 are significantly different from dolichofacial patients who yielded a mean AHI value of 10.52. This finding disagrees with the results commonly found in the literature which does not establish a strong association between dolichofacial patients and OSA.^6^^,^ 7 ^,^ 9 ^,^ 10 ^,^ 22 However, this fact is not unanimous;^8^^,^ 23 it is supported by Grauer et al^20^ who used cone-beam computed tomography and did not find any differences in airspace volume for dolichofacial patients. Moreover, the study by Haskell et al^23^assessed 50 patients by means of cone-beam computed tomography and found greater airspace in vertical patients.

With a view to highlighting the importance of the factors assessed herein (facial pattern and type), multiple linear regression analysis was employed to assess the relevance of the present variables in comparison to other OSA predicting factors, such as obesity mathematically measured by BMI.^4^^,^ 10 ^,^ 11 ^,^ 21 In the present analysis, Pattern II patients had the AHI value increased in 6.98 episodes of apnea per hour of sleep when compared to Pattern I individuals. Conversely, Pattern III patients had the AHI value reduced in 11.45 when compared with Pattern II patients. Data analysis suggests that while Pattern II might render OSA more severe, Pattern III seems to protect patients against the sleeping disorder. This statistical analysis did not reveal any relationship between facial type and OSA.

The low prevalence of long face (4.0%) and short face (3.6%) in the sample studied did not allow us to make further inference with regard to the occurrence of OSA in either one of these morphological groups. Likewise, low prevalence also seems to be found in the overall population. In 2007, the study conducted by Siécola^24^ comprised 151 children aged between 7 and 13 years old and who were enrolled in two different schools in the city of Bauru. The study found a prevalence of 5.96% of long-face individuals and 1.98% of short-face individuals. Nevertheless, should the morphological diagnosis of long face be strongly associated with OSA, as suggested by a few studies,^6^^,^ 7 ^,^ 9 ^,^ 10 ^,^ 22 this group would be expected to appear in a higher number in a sample selected at a clinics specialized in sleeping disorders where 69.5% of individuals had OSA.

Due to being a multifactorial disease, the etiology of OSA is too diverse and complex to be explained by a simple relationship established between facial morphology and the development of the disease. However, therapeutic orthodontic actions should respect the tendency towards these results and include functional aspects related to OSA in their therapeutic practice. The importance of considering patients' craniofacial morphology as a factor that protects or worsens OSA is confirmed by asystematic literature review carried out by Pirklbauer et al^15^ in 2011. The authors found that the most effective surgical approach employed to treat OSA is advancement of the maxilla and mandible, i.e., exactly when a drastic facial morphological change occurs. Post-operative polysomnography results can be compared to those yielded by CPAP therapy.

Adult brachyfacial patients and Pattern II due to mandibular deficiency should be included in the benefits of decompensatory orthodontic treatment for surgeries of mandibular advancement: a functional breathing benefit that widens the upper airways while reducing the anatomical risk of OSA development. Meanwhile, if patients comprising this diagnostic group have incomplete growth, compensatory treatment with reduction in intraoral volume, such as upper premolars extraction, should be avoided, as it could result in maintenance or exacerbation of anatomical disadvantages likely to lead to the development of OSA in the long term, particularly when associated with other predisposing factors such as obesity and aging.^1^^,^ 3

Adult Pattern III patients, however, should have mandible position preserved, in order to avoid potential mandibular setback, unless supported by prognathism severity and impaired esthetics. Thus, whenever recommending advancement of the maxilla, clinicians should consider including the benefit of widening upper airways in patients' orthodontic and surgical planning, thereby preserving the functional benefits these patients seem to have with regard to the development of OSA. For growing patients, the protocol of widening the maxilla is also supported by the gain in breathing volume, which minimizes the potential for developing OSA, thereby providing patients with undeniable functional benefits.

The evidence of an association between facial morphology and OSA point to therapeutic orthodontic modalities that preserve or enhance the shape of the anatomical traits of the face, even though such gain is limited by genetic determinism. This approach would aim at offering long-term functional respiratory, yet minimal, benefits.

## CONCLUSIONS

Based on the results of this study, it is reasonable to conclude that:

1) Pattern II seems to worsen OSA, whereas Pattern III seems to decrease its severity;
2) Brachyfacial type was more associated with severe apnea than the dolichofacial type;
3) The following aspects influence AHI in an ascending order: facial morphological pattern, sex, age and BMI.
